# Prediction of residual astigmatism in cataract surgery at different diameter zones using optical biometry measurement

**DOI:** 10.1038/s41598-022-08253-6

**Published:** 2022-03-11

**Authors:** Yin-Hsi Chang, Christy Pu, Ken-Kuo Lin, Jiahn-Shing Lee, Chiun-Ho Hou

**Affiliations:** 1 Department of Ophthalmology, Chang Gung Memorial Hospital, Linkou Medical Center, No. 5, Fuxing Street, Guishan District, Taoyuan, 33305 Taiwan, ROC; 2 Institute of Public Health, School of Medicine, National Yang Ming University, Taipei, Taiwan; 3 Department of Medicine, College of Medicine, Chang Gung University, Taoyuan, Taiwan; 4 Department of Ophthalmology, National Taiwan University Hospital, Taipei, Taiwan

**Keywords:** Corneal diseases, Lens diseases, Outcomes research

## Abstract

The studies for astigmatism prediction error at different diameters using optical biometry are scant. We investigated patients who underwent cataract surgery with monofocal, nontoric intraocular lens (IOL) from 2017 through 2019 in a medical center. Patients with prior refractive surgeries, corneal opacity, or surgical complications were excluded. Corneal astigmatism (CA) was measured using AL-Scan at 2.4- and 3.3-mm diameter zones and calculated using the Barrett toric calculator preoperatively and postoperatively. The mean absolute error and centroid prediction error for the two zones were computed using double-angle plots. In total, 101 eyes of 76 patients were analyzed. Mean patient age was 68.7 ± 9.3 years and mean preoperative CA power was 0.7 ± 0.5 D. The overall centroid prediction error a 3.3 mm (0.09 ± 0.58 D@25) was significantly lower than that at 2.4 mm (0.09 ± 0.68 D@87) on the X-axis (*P* = 0.003). The 3.3-mm measurement also had a lower centroid prediction error than the 2.4-mm did for eyes with against-the-rule (ATR) and oblique astigmatism (P = 0.024; 0.002 on X-axis, respectively). The 3.3-mm measurement provided a more accurate CA estimation than the 2.4-mm did, particularly for ATR astigmatism. Diameter zone and astigmatism type should be considered crucial to precise astigmatism calculation.

## Introduction

Cataract surgery has been developed into a refractive surgery that corrects both spherical and astigmatic errors^1^. The use of premium intraocular lenses (IOLs), such as toric IOLs, has gained popularity for minimizing refractive error over the past years^2,3^. The mean preexisting corneal astigmatism (CA) was 1.06 D in a large cross-sectional study, in which 20% of cataract-operated eyes had CA of at least 1.5 D^4^. Residual astigmatism after cataract surgery was a common refractive error observed in a significant proportion of patients^5^. The amount of residual astigmatism could be significantly reduced to 0.17–0.77 D with toric IOL implantation under appropriate calculation^3^. Therefore, accurate measurement and preexisting CA calculation are essential for surgical planning to achieve the desired refractive outcome^6^.

Over the past decades, various devices and calculators have been applied for optimal CA predictive outcome. For posterior corneal astigmatism (PCA), the Barrett toric calculator has achieved precise results and is widely recognized as a useful calculation method^7–10^. Studies have compared the keratometrics (K) of different measuring devices combined with the Barrett toric calculator, and the predictive accuracy was found to vary between devices^11,12^. Each device utilizes a different methodology, with differences in the operating principle, wavelength for machinery design, and diameter zones for measurement. Based on the literature, determining whether machinery design or diameter zone contributes to the predictive accuracy is challenging. Previous studies have only compared the agreement and repeatability of devices^13–18^.

Measuring diameter zones for the cornea vary between devices. For instance, Auto Kerato-Refractometer (Topcon Co., Ltd.) takes K measurements at a diameter zone of approximately 3.3 mm; IOLMaster (Carl Zeiss Meditec AG) measures at a 2.5-mm diameter zone based on six reference points in a hexagonal pattern^19^; Lenstar (Haag-Streit AG) has 32 measurement points on two concentric rings with diameters of 1.65 and 2.3 mm^20,21^; AL-Scan (Nidek, Co., Ltd.) provides two sets of K measurements at 2.4- and 3.3-mm diameter zones^22^; and Pentacam (Oculus Optikgeräte GmbH) measures at 3 mm and adjusts the recorded zones from 1 to 8 mm with a rotating Scheimpflug camera^23^. The diameter zone may also play a role in the accuracy of CA measurement and prediction. If the same instrument such as Pentacam or AL-Scan measures two or more optical zones, the variation resulting from instrument design could be eliminated. The diameter zone becomes the only determining factor. A previous study in patients receiving refractive surgery using Pentacam showed that the cylinder power varied between zones^24^. However, no study has compared optical biometry in cataract surgery.

The present study aimed to evaluate and compare the CA prediction error at different diameter zones with measurement conducted using an optical biometry AL-Scan. AL-Scan employs a 970-nm light-emitting diode (LED) for keratometry by projecting (360°) double-mire rings at diameters of 2.4 and 3.3 mm; these two sets of K measurements are obtained simultaneously. To the best of our knowledge, this is the first study to evaluate the role of measurement zones with optical biometry in patients receiving cataract surgery.

## Methods

### Patients

This study retrospectively reviewed the medical records of patients who underwent cataract surgery with monofocal, nontoric IOL implantation performed by a single surgeon (C.H.H.) at Chang Gung Memorial Hospital, Taipei, from December 2017 through December 2019. Patients with complete pre- and postoperative AL-Scan measurements were enrolled. A minimum of 1-month postoperative follow-up with postoperative best corrected visual acuity (BCVA) of 20/30 or higher was required. Patients with BCVA < 20/30 were excluded because these data may not reflect accurate readings of subjective astigmatism. Patients with intraoperative or postoperative complications resulting in IOL decentration or malposition, such as posterior capsular tear, residual cortex or epi-nucleus, zonular dialysis, and severe capsular fibrosis, were excluded^25^. Other exclusion criteria were corneal opacity, history of other refractive or intraocular surgeries that may contribute to astigmatism, and variability or instability in K readings. The study was performed in accordance with the tenets of the Declaration of Helsinki and was approved by the Institutional Review Board of Chang Gung Memorial Hospital (no. 202000192B0C601), which waived the requirement of written informed consent.

### Surgical procedure

All cataract surgeries were performed via a 2.65-mm superotemporal or superonasal clear corneal incision (at 145°). After routine cataract extraction by phacoemulsification and cortex removal, a monofocal, nontoric, acrylic, foldable IOL was inserted in the capsular bag using an injector. The clear corneal wound was sealed by stromal hydration with an irrigation cannula.

### Measuring device

Nidek AL-Scan (Nidek Co., Ltd.) is a partial coherence interferometry-based optical biometer that measures the axial length by using an 830-nm infrared laser diode^15^. It also uses a 970-nm light-emitting diode for K measurements by projecting (360°) double mire rings at diameters of 2.4 mm and 3.3 mm, which are reflected from the corneal surface. It provides these two sets of K measurements simultaneously. All K values, representing the curvature of the steep and flat meridians, are presented in diopters (D). Other parameters including the anterior chamber depth, central corneal thickness, white-to-white distance, and pupil distance can also be obtained using AL-Scan. In addition, corneal irregularity was analyzed using Pentacam (Oculus Optikgeräte GmbH). Pentacam is a rotating Scheimpflug camera for anterior segment analysis. It measures topography and corneal power based on multiple cross-sectional images along the optical axis^23^.

### Method of calculation

The online Barrett toric calculator v2.0 (https://www.apacrs.org/) was used with both 2.4- and 3.3-mm K readings from AL-Scan to estimate the total CA regarding the predicted PCA. The mean difference between the preoperative and postoperative CA, namely the surgically induced astigmatism (SIA), was calculated and applied in preoperative CA estimation. Moreover, postoperative corneal measurement was used for CA prediction, and these K readings were obtained postoperatively after the corneal incision was made. In this case, SIA data were not considered in calculations. To precisely calculate CA, patients with nontoric IOL implantation were included. Lenticular astigmatism was eliminated in such patients, and the cornea almost became the only optical component in the eye contributing to astigmatism^26^. The refractive astigmatism was assumed to represent the total CA in these eyes postoperatively.

### Accuracy determination

Double-angle plots were used to display the magnitude and axis or meridian of the average astigmatism (https://ascrs.org/tools/astigmatism-double-angle-plot-tool)^27^. We selected double-angle plots instead of the Alpins method because both methods share the same concept of double-angle vector diagrams, and double-angle plots have been adopted extensively in recent years, as the gold standard method for astigmatism analysis for intraocular lens-based surgery^27^. This tool could also be used to calculate the refractive astigmatism prediction errors, centroid values, standard deviations (SDs), and 95% confidence ellipses of the dataset and of the centroid, which is the vectoral center of the data. This method considers both astigmatism magnitude and axis, whereas the mean value only reflects the magnitude. Therefore, the centroid prediction error is preferred over the mean absolute error for astigmatic outcomes in cataract surgery^28^. The corneal plane was used to represent refractive astigmatism. For left eyes, the angle of the axis of astigmatism was converted using the formula suggested by Kawahara et al. (transformed angle = 180 − angle)^29^. Vector analysis was used to compared astigmatism values between the orthogonal X and Y components according to the method by Holladay et al.^30^. The mean of a set of X or Y values was calculated independently. In addition, astigmatism types were classified according to the steep meridian orientation as with-the-rule (WTR), against-the-rule (ATR), and oblique, as measured by AL-Scan (60°–120° for WTR astigmatism, 0°–30° or 150°–180° for ATR astigmatism, 30°–60° or 120°–150° for oblique astigmatism). The mean absolute error and centroid errors were also calculated for each subgroup.

### Statistical analysis

Statistical analyses were conducted using Stata software, v15 (StataCorp LLC, College Station, Texas, USA). Paired t tests were performed to compare the measurements of two diameter zones in the same patient. Multiple linear regression analysis was used to explore the possible factors influencing prediction error, such as age, sex, axial length, astigmatism type, and corneal irregularity. All values were expressed as mean ± SD. A *p* value of < 0.05 was considered statistically significant.

### Results

**Figure 1:**
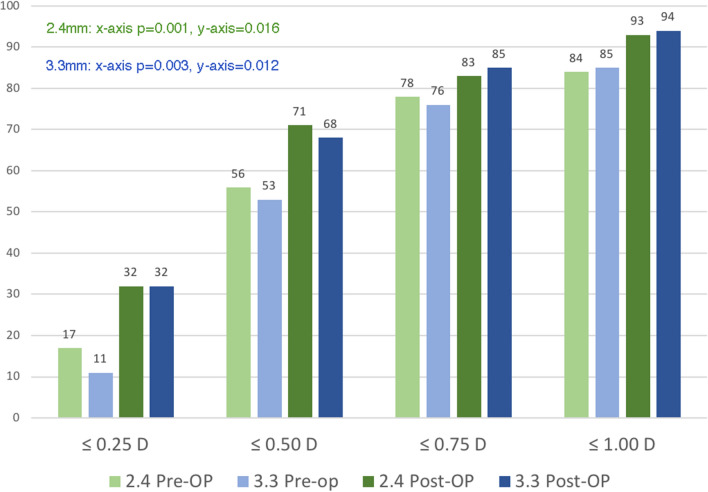
Distribution of astigmatism prediction error. Using postoperative keratometrics for calculation reduces prediction error compared with using preoperative keratometrics considering surgically induced astigmatism. More than 90% of the patients have a prediction error of < 1.0 D with postoperative keratometrics. This trend is observed in both the 2.4- and 3.3-mm groups. *D* diopter, *pre-op* preoperative, *post-op* postoperative.

**Figure 2:**
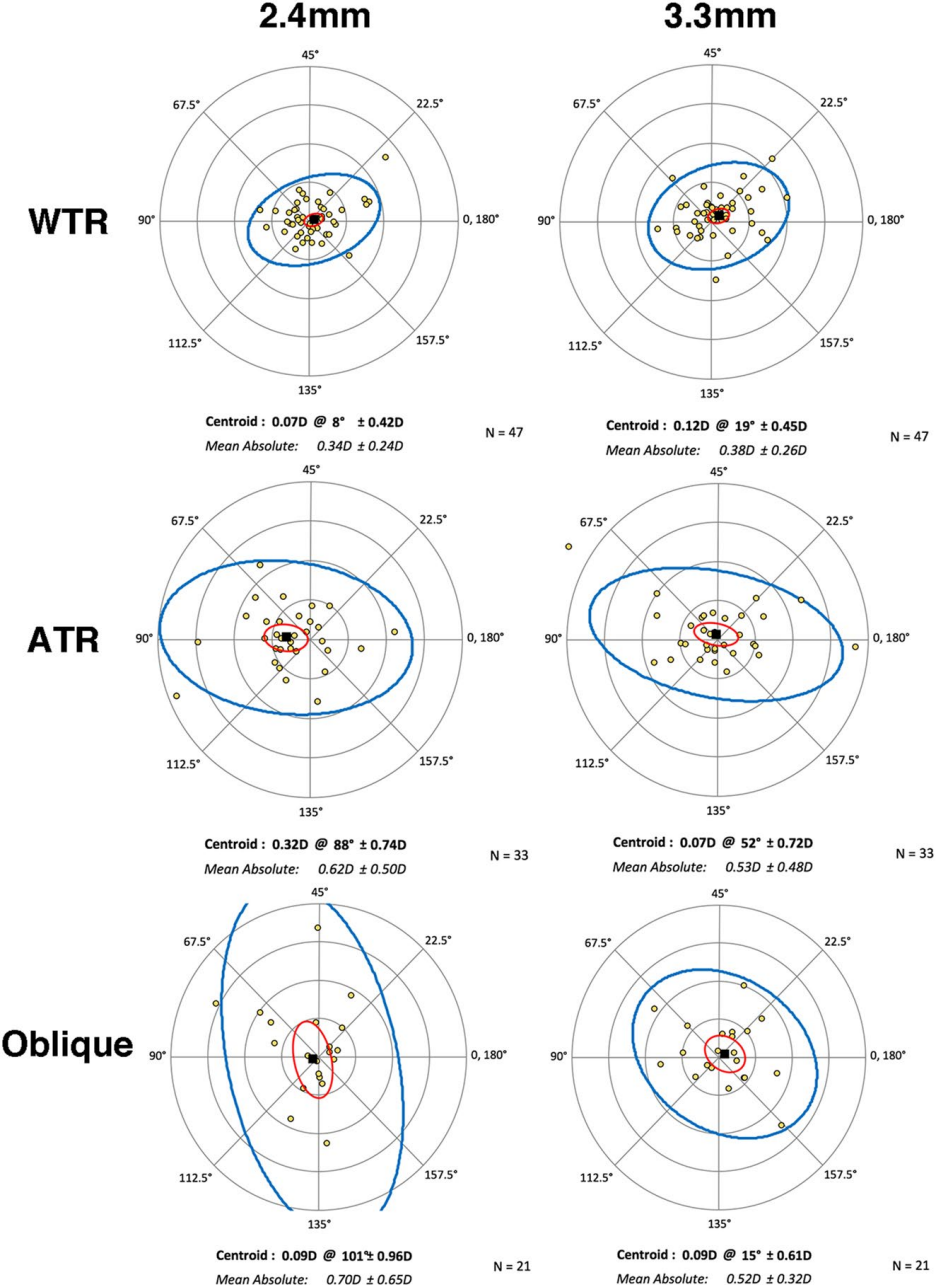
Double-angle plots of the astigmatism prediction error by diameter zone and astigmatism subgroup. The red and blue lines represent the 95% confidence ellipses of the centroid and dataset, respectively. These two 95% confidence ellipses are apparently smallest in the WTR subgroup. *WTR* with-the-rule, *ATR* against-the-rule.

A total of 141 cataract surgeries with nontoric IOL implantation with completed measurement protocol were performed uneventfully by C.H.H. at Chang Gung Memorial Hospital, Taipei, during the study period. In total, 38 eyes did not achieve a postoperative BCVA of 20/30 because of macular edema, underlying retinopathy, or corneal opacity. Two eyes with a history of laser in-situ keratomileusis were excluded. Finally, 101 eyes of 76 patients were included in the study. Patient baseline characteristics are shown in Table 1. Approximately 99% of the patients were aged > 50 years. The majority of the patients had astigmatism of < 2.0 D based on AL-Scan measurements at both optical zones. Approximately 56.4% had WTR astigmatism. The platforms for IOL implantation included Tecnis, NIDEK, and enVista. Table 2 shows the preoperative and postoperative CA magnitudes measured with 2.4- and 3.3-mm diameter zones, respectively. The mean absolute CA was not significantly different between the two diameter zones preoperatively (*P* = 0.3071). The astigmatism prediction error decreased when using postoperative keratometry compared with that when using preoperative measurement considering SIA data (2.4 mm X-axis: *P* = 0.001; 2.4 mm Y-axis: *P* = 0.016; 3.3-mm X-axis: *P* = 0.003; 3.3-mm Y-axis: *P* = 0.012) (Fig. 1). The mean absolute prediction error between these two zones was nonsignificant (*P* = 0.799). The centroid prediction error was 0.09 ± 0.68 D @87 and 0.09 ± 0.58D @25 at optical diameters of 2.4 and 3.3 mm, respectively. Vector analysis of the X component revealed that the centroid prediction error was significantly lower at an optical diameter of 3.3 mm (0.058 ± 0.511 D) than for 2.4 mm (− 0.092 ± 0.489 D; X-axis: *P* = 0.003; Table 3). Additionally, a significantly lower centroid prediction error at an optical diameter of 3.3 mm than for 2.4 mm was also observed in the ATR (X-axis: *P* = 0.024) and oblique (X-axis: *P* = 0.002) subgroups. The centroid prediction error was significantly lower at an optical diameter of 2.4 mm (0.07 ± 0.42 D@8) compared with that at 3.3 mm (0.12 ± 0.45 D@19) in the WTR subgroup (Y-axis: *P* = 0.031; Table 3). Double-angle plots revealed the spatial distribution of the postoperative astigmatic prediction errors (Fig. 2). In the exploration of possible factors affecting prediction errors, age, sex, AL, IOL power, preoperative astigmatism, and corneal irregularity did not cause significant differences in the univariate and multivariate linear regression analyses (Supplement 1).

**Table 1:** Patient’s baseline characteristics.

Parameter	Total
Patient number	101
Age (mean)	68.7 ± 9.3 (47–89)
Gender (male:female)	53 (52.5%):48 (47.5%)
Laterality (OD:OS)	56 (55.4%):45 (44.6%)
Axial length (mm)	24.7 ± 2.0 (21.86–30.25)
IOL power (D)	18.1 ± 5.2 (6–27.5)
Pre-operative cylinder power (D)	0.7 ± 0.5 (0.06–2.03)
Astigmatism type (WTR:ATR:oblique)	47 (46.5%):33 (32.7%): 21(20.8%)

*ATR* against-the-rule, *D* diopters, *IOL* intraocular lens, *OD* right eye, *OS* left eye, *WTR* with-the-rule.

**Table 2:** Pre-operative and post-operative corneal power measured with AL scan at 2 diameter zones.

Parameter	Pre-OP			Post-OP		
	2.4-mm	3.3-mm	p value	2.4-mm	3.3-mm	p value
Average K (D)	43.92 ± 0.35	43.88 ± 0.35	0.002	44.05 ± 0.4	43.97 ± 0.37	< 0.001
Flattest K (D)	43.57 ± 1.5	43.54 ± 1.49	0.075	43.65 ± 1.49	43.6 ± 1.49	0.07
Steepest K (D)	44.27 ± 1.6	44.23 ± 1.6	0.023	44.45 ± 1.61	44.35 ± 1.57	0.01
Mean absolute CA (D)	0.70 ± 0.43	0.69 ± 0.44	0.3071	0.80 ± 0.51	0.75 ± 0.47	0.258
**N of patients**						
≤ 0.50 D	42	43		33	37	
≤ 1.00 D	76	79		74	75	
≤ 1.50 D	93	95		90	92	
≤ 2.00 D	101	100		99	99	

*CA* corneal astigmatism, *D* diopters, *K* keratometry, *No.* number, *Pre-OP* pre-operative, *Post-OP* post-operative.

**Table 3:** Comparison of post-operative residual astigmatism prediction error at 2 diameter zones and subgroup analyses.

Parameter(D)	2.4 mm	3.3 mm	P value
**Total (n = 101)**			
Mean ± SD	0.51 ± 0.46	0.46 ± 0.36	p = 0.799
Centroid ± SD	0.09 ± 0.68 @87	0.09 ± 0.58 @25	p = 0.003*(x-axis); 0.123 (y-axis)
**WTR (n = 47)**			
Mean ± SD	0.34 ± 0.24	0.38 ± 0.26	p = 0.250
Centroid ± SD	0.07 ± 0.42 @ 8	0.12 ± 0.45 @ 19	p = 0.485 (x-axis); 0.031* (y-axis)
**ATR (n = 33)**			
Mean ± SD	0.62 ± 0.50	0.53 ± 0.48	p = 0.413
Centroid ± SD	0.32 ± 0.74 @88	0.07 ± 0.72 @ 52	p = 0.024* (x-axis); 0.479(y-axis)
**Oblique (n = 21)**			
Mean ± SD	0.70 ± 0.65	0.52 ± 0.32	p = 0.934
Centroid ± SD	0.09 ± 0.96 @101	0.09 ± 0.61 @15	p = 0.002* (x-axis); 0.573(y-axis)

### Discussion

For astigmatism prediction in cataract surgery, the centroid error includes both the magnitude and axis for analysis, which is preferred over the mean absolute error^28^. In our study, the difference between the 2.4- and 3.3-mm diameter zones was nonsignificant when the mean absolute prediction error was used, but it was significant when the centroid prediction error was used. We believe that the difference was due to the combination of astigmatism magnitude and axis. In summary, the 3.3-mm diameter zone measurement in optical biometry produced a more accurate estimation of astigmatism than for 2.4-mm, particularly for ATR astigmatism, regardless of age, sex, axial length, IOL power, preoperative astigmatism, and corneal irregularity. Moreover, postoperative K provided better accuracy for prediction error than the preoperative K considering SIA. To the best of our knowledge, this is the first study to evaluate the effect of measurements with different diameter zones using the same optical biometry instrument on the prediction of astigmatism after cataract surgery.

Several studies have compared the prediction errors of different measuring machines and diameter zones. Although the results were conflicting, some studies have suggested that measurements at paracentral zones close to 3.3 mm were more accurate than those at smaller diameter zones. The paracentral 4-mm zone measurement with Pentacam was more accurate than the central 2.5-mm zone measurement with IOLMaster in a study by Park et al.^8^ Another article showed more precise results at the 3.3-mm zone with AutoKM with a centroid error of 0.21 ± 0.45 D@45.9 compared with 0.24 ± 0.37 D@68.7 at the 2.5-mm zone mm with IOLMaster and 0.49 ± 0.85 D@73.0 within the central 2.0 mm with Galilei^7^. On the other hand, some articles have demonstrated the central zone to be more accurate than the paracentral zone, even though the measuring machine was different. For example, the centroid error was lower within the 1.65- and 2.3-mm measurement zones with Lenstar than at the 2.5-mm zone with IOLMaster^11,12^. Lenstar also exhibited lower centroid error than Cassini at 2.0, 3.0, and 4.0 mm and Pentacam at 4 mm^9^. The centroid errors were 0.07 ± 0.28 D@160, 0.10 ± 0.44 D@156, and 0.23 ± 0.56 D@158 for Lenstar, Cassini, and Pentacam, respectively. However, these studies have compared distinct measuring devices including biometry and topography, and the device used for measuring at smaller diameter zones was Lenstar. The difference may be attributed to not only the optical zones but also the diverse machinery design as well as variable calculation with or without Barrett toric calculation. Due to the inconsistency in previous results and diversity in study design, the optimal diameter zone for measurement was not conclusive.

We utilized AL-Scan in our analysis because it provided K measurements at different diameter zones with the same device, eliminating the influence of machinery design. Our results were consistent with those of previous studies: the 2.4-mm diameter zone showed a steeper KM than the 3.3-mm diameter zone among cataract patients^14,15,17,31^. The KM difference between the 2.4- and 3.3-mm zone with AL-Scan was 0.01–0.05 D in those studies, whereas that in our patients was 0.04 D preoperatively and 0.08 D postoperatively. The corneal curvature was steeper in the center and flatter in the periphery, giving a prolate shape; therefore, it was reasonable that the corneal power differed between optical zones^32^.

Pentacam is another device for measuring CA at different zones concurrently^24,33^. Dong et al. used total corneal refractive power (TCRP) on Pentacam to compare CA at 3- and 4-mm zones in cataract patients preoperatively^33^. The centroid error was 0.82 ± 2.13 D@2 and 0.67 ± 2.07 D@178, respectively, suggesting that corneal power and astigmatism data differed based on diameter zones. However, this study did not provide prediction accuracy for these diameter zones. Another Pentacam study indicated a trend of increasing CA from the central cornea to the peripheral cornea but did not provide a comparison of measured corneal astigmatism with subjective refraction for determining prediction errors^24^. Also, the study subjects were young adult candidates for corneal refractive surgery aged 26.3 ± 6.6 years. They had low to moderate astigmatism ranging from 0 to 3.7 D, which was different from all other residual astigmatism studies enrolling older patients undergoing cataract surgery, and the lenticular astigmatism was not eliminated. Our study showed both preoperative CA and postoperative centroid prediction error in the presence of IOL, which was more accurate in analyzing CA.

In the subgroup analysis of astigmatism types, centroid prediction error was smaller at 3.3 mm compared with that at 2.4 mm in both ATR and oblique astigmatism eyes. By contrast, the centroid prediction error was smaller at 2.4 mm compared with that at 3.3 mm in eyes with WTR astigmatism. Two studies comparing the prediction error at different diameter zones among astigmatism types have used Pentacam TCRP as the paracentral 4-mm measurement. Park et al. recorded measurements at 2.5 mm with IOLMaster, whereas Ribeiro et al. applied the 1.65- and 2.3-mm zones with Lenstar for the central zone measurement^8,9^. Park et al. demonstrated that the centroid error at the paracentral zone (0.29 ± 0.84 D@50) was smaller than that at the central zone (0.43 ± 0.87 D@3) in ATR eyes, and the present results were in agreement with these findings^8^. By contrast, Ribeiro et al. provided opposite findings related to ATR eyes; the paracentral zone produced a larger centroid error (0.27 ± 0.48 D@179) than did the central zone (0.01 ± 0.25 D@167)^9^. However, WTR eyes had a smaller centroid error (0.09 ± 0.32 D@155) at the centroid zone compared with that at the paracentral zone (0.39 ± 0.61 D@177), as demonstrated in our study^9^. Therefore, the type of astigmatism could influence prediction accuracy and should be considered during analyses. Moreover, these two studies analyzed cataract patients with toric IOL implantation, and the conversion of lenticular astigmatism with different effective lens position to the corneal plane could be another influencing factor. Additionally, neither of the studies included an oblique astigmatism subgroup, so no comparison could be made. More studies with all astigmatism types would be needed to explain these findings in the future.

Clinically, the ophthalmologists chose either 2.4- or 3.3-mm K on AL-Scan based on their own preference to calculate IOL power and astigmatism. Our results provided a reference in which the optical zone was more accurate. This can be helpful when using optical biometry AL-Scan for preoperative planning in cataract refractive surgery. However, the difference was small and may not be perceivable by patients. According to the literature, the sensitivity threshold for astigmatism is approximately 0.20 to 0.30 D^34^, and a change of 0.25 D is measurable in clinical settings following cataract surgery^35^. Another study that used visual stimulation reported that the cutoff point for perceptible cylindrical errors was approximately 0.15 D^36^. In our study, when the total number of eyes was included, the centroid prediction error was < 0.15 D at diameters of 2.4 and 3.3 mm. However, in our subgroup analysis of astigmatism type, the differences between diameters of 2.4 and 3.3 mm were greater in the ATR group (0.32 vs 0.07 D) than in the other groups. These results indicated that the difference in cylindrical error might be more noticeable by patients with ATR astigmatism. We hope this finding can be incorporated into machinery design and as a reference for IOL formula assessments.

Our study had some limitations. First, it was a retrospective single-center study. Selection bias is possible due to the retrospective nature of the study, but it was minimized through consecutive sampling. Second, many factors affect the accuracy of refractive astigmatism measurement. For example, the corneal curvature could change with accommodation. To counter this, each measurement was taken under the same setting^26^. Tear film instability may cause variation in astigmatism magnitude and axis. Therefore, lubricants were added at least 2 weeks prior to the measurement if the patient had dry eyes. Third, tilting or decentration of the implanted IOL may contribute to the astigmatism. Even in uneventful cataract surgery, the mean IOL tilt was 1.54° and the decentration was 0.21 mm^37^. However, the differences contributing to astigmatism were thought to be minor. Fourth, underestimation or overestimation of SIA can also lead to prediction errors. Therefore, we used postoperative K values to eliminate SIA effects. In studies using preoperative corneal astigmatism combined with SIA for prediction error calculation, the follow-up period must be 2 to 6 months after surgery, when corneal power has become stable^38,39^. Finally, only patients with low astigmatism were studied, and caution should be exercised in drawing inferences to patients with high astigmatism.

The accuracy of residual astigmatism prediction may be influenced by different measuring methods and diameter zones. For diameter zones, using AL-Scan, we obtained smaller centroid prediction errors in 3.3-mm K readings than in 2.4-mm readings, particularly in eyes with ATR astigmatism. As advanced refractive cataract surgery becomes increasingly widespread, the measuring diameter zone should be considered in precise astigmatism calculation to ensure optimal refractive outcome. Future studies of patients with medium to high astigmatism and of application in premium toric IOL implantation are needed.

## Supplementary Information

Supplementary Table 1.
