# Supplementary material for: Prediction of residual astigmatism in cataract surgery at different diameter zones using optical biometry measurement
Source: Sci Rep. 2022 Mar 11;12:4305. doi: 10.1038/s41598-022-08253-6 (PMC8917119; doi:10.1038/s41598-022-08253-6)
Supplement: Supplementary file 1 — Supplementary Table 1. [file 41598_2022_8253_MOESM1_ESM.docx]

**Supplement**: Linear Regression Analyses of Variables Associated with Centroid Prediction Error

|  | Univariate analysis | |  | Multivariable analysis | |
| --- | --- | --- | --- | --- | --- |
| Variables | *B* (95% CI) | *P* |  | *B* (95% CI) | *P* |
| Age | X -0.006 (-0.017, 0,004)  Y 0.002 (-0.005, 0.009) | 0.226  0.514 |  | X -0.008 (-0.022, 0.006)  Y 0.002 (-0.007,0.011) | 0.258  0.588 |
| Sex |  |  |  |  |  |
| Female | Reference |  |  | Reference |  |
| Male | X -0.072 (-0.266, 0.112)  Y 0.061 (-0.065, 0.186) | 0.463  0.341 |  | X -0.094 (-0.297, 0.109)  Y 0.081 (-0.052, 0.215) | 0.361  0.229 |
| Axial length | X 0.013(-0.035, 0.061)  Y -0.011 (-0.042, 0.020) | 0.600  0.494 |  | X -0.078 (-0.211, 0.055)  Y -0.013 (-0.101, 0.075) | 0.249  0.774 |
| IOL power | X- 0.010(-0.028, 0.009)  Y 0.004 (-0.008, 0.016) | 0.296  0.493 |  | X -0.033 (-0.083, 0.017)  Y <-0.001 (-0.033, 0.033) | 0.189  0.997 |
| Pre-op astigmatism | X -0.045(-0.241, 0.151)  Y 0.005 (-0.122, 0.132) | 0.650  0.936 |  | X -0.024 (-0.235, 0.187)  Y -0.012 (-0.151, 0.126) | 0.822  0.860 |
| Corneal irregularity | X 0.578(-5.474, 6.630)  Y -0.242(-4.174, 3.689) | 0.850  0.903 |  | X 2.844 (-3.932, 9.621)  Y -1.383 (-5.844, 3.077) | 0.407  0.540 |
